# the specificity of female addictions in morocco (A cross-sectional analytical study)

**DOI:** 10.1192/j.eurpsy.2023.1373

**Published:** 2023-07-19

**Authors:** F. Z. Chamsi, H. Abrebak, O. Radouane, A. EL ammouri

**Affiliations:** psychiatry department, university hospital tangier morocco, tangier, Morocco

## Abstract

**Introduction:**

Addiction is a chronic disorder resulting in physical, psychological, and/or social harm following the repetitive, compulsive consumption of a substance. In Morocco, there are few data on female addictive behavior, apart from those concerning the prevalence of consumption of products based on surveys of the general population.

**Objectives:**

Report the current epidemiological, clinical, and therapeutic peculiarities of female addiction according to a heuristic approach (based on proven scientific evidence).

**Methods:**

A questionnaire was distributed to patients followed at the addiction center in Tetouane and the Ar-razi psychiatric hospital in Tangier. We also carried out a systematic review of the literature, focusing on about ten articles. The main search engines are PubMed, Medline, Science Direct.

**Results:**

The substance use disorder concerns cannabis in 68% of cases, tobacco in 52.1% of cases, alcohol in 40.33% of cases, benzodiazepines in 33.61%, cocaine in 15.96%, opioids (7.5%), and inhalants (1.6%). 73.8% of residents have psychiatric comorbidities, of which 42.9% suffer from depression, 13.40% from bipolar disorder, 10% from generalized anxiety disorder, 4, 20% have panic disorder, 1.7% have social anxiety, 0.8% have post traumatic stress syndrome and 0.8% have bulimia. 58.8% of patients had a personality disorder, 36.1% attempted suicide, 16% suffered physical violence, and 20.20% of patients were victims of rape.

International literature shows the growing interest in gender issues in the field of addictions (whether they involve the consumption of legal or illegal products or behaviors such as gambling, food, purchases, sexuality, etc.), apart from those concerning the prevalence of consumption of general population survey products, shows the growing interest in gender issues in the field of addictions. This problem has been recognized as being of crucial importance and has been posed as a priority objective

**Conclusions:**

Minimum levels of consumption among women tend to obscure the impact of gender relations on female addicts. Through this research, we hope to identify the differences between male and female addictions. The overdetermination of certain forms of psychiatric suffering and/or certain living conditions makes women more vulnerable to the problematic use of psychotropic drugs. On the other hand, women have to gain from being able to benefit from specific responses within the framework of front-line or support and care systems.

**Disclosure of Interest:**

None Declared

